# Lateral luxation: Is root resorption an unavoidable complication?

**DOI:** 10.1002/ccr3.5880

**Published:** 2022-05-18

**Authors:** Ines Kallel, Malek Lagha, Eya Moussaoui, Nabiha Douki

**Affiliations:** ^1^ Department of Dental Medicine Hospital Sahloul Sousse Tunisia; ^2^ Faculty of Dental Medicine University of Monastir Tunisia; ^3^ Laboratory of Research in Oral Health and Maxillo Facial Rehabilitation (LR12ES11) University of Monastir Tunisia

**Keywords:** complication, lateral luxation, root resorption, traumatology

## Abstract

Lateral luxation is defined as a traumatic displacement of a tooth in any direction other than axially. A laterally luxated tooth is often immobile because of its bony lock. It produces a high metallic sound during percussion. Pulp sensibility testing is likely to be negative during the initial follow‐up. However, the tooth should be monitored until a definitive pulp diagnosis is made. Treatment includes local anesthesia, suturing soft tissue injuries, manual repositioning of the luxated tooth, and stabilization with a flexible splint for 4 weeks. Given the risk of pulp necrosis following lateral luxation, particularly in teeth with radiographically closed apices and severe displacement, an immediate (prophylactic) root canal treatment is recommended. The objective of this work was to report and discuss the management of a case of lateral luxation involving a permanent tooth treated by reduction, followed by contention with 0.4 steel wire and composite resin, and endodontic treatment, and which was complicated by root resorption 3 months later. We also highlighted the possible complications following lateral luxation, especially root resorption.

## Abstract 2

The key clinical message of this case report is to detail the management of lateral luxation and to highlight the importance of clinical and radiological follow‐up in order to detect the possible complications, particularly root resorption.

## 1 INTRODUCTION

Dental luxation constitutes one of the most severe traumatic injuries affecting the teeth and the surrounding tissues. It is the result of the impacts of different intensities and directions causing displacement of the tooth or teeth from their normal position to a greater or lesser degree. Lateral luxation of permanent teeth is a common injury accounting for 23.3% of dental injuries.^1^ The periodontal tissue is damaged in approximately 26% of laterally luxated teeth.^2^ However, its prognosis is much better than that of other dental displacement traumas.^3^ Generally, in this type of injury, the vascular supply is affected. The pulp has a certain capacity to survive, but it is a limited capacity. In severe displacement, the vasculature to the pulp is interrupted, and as a consequence, the pulp becomes necrotic. Root resorption is the most concerning complication because it can lead to tooth loss. Furthermore, this complication can arise years after treatment, emphasizing the importance of long‐term follow‐up.^4^

The purpose of this paper was to present a case of lateral luxation of permanent maxillary incisors, to provide the proper treatment, and to highlight the possible complications, especially root resorption.

## 2 CASE REPORT

**FIGURE 1:**
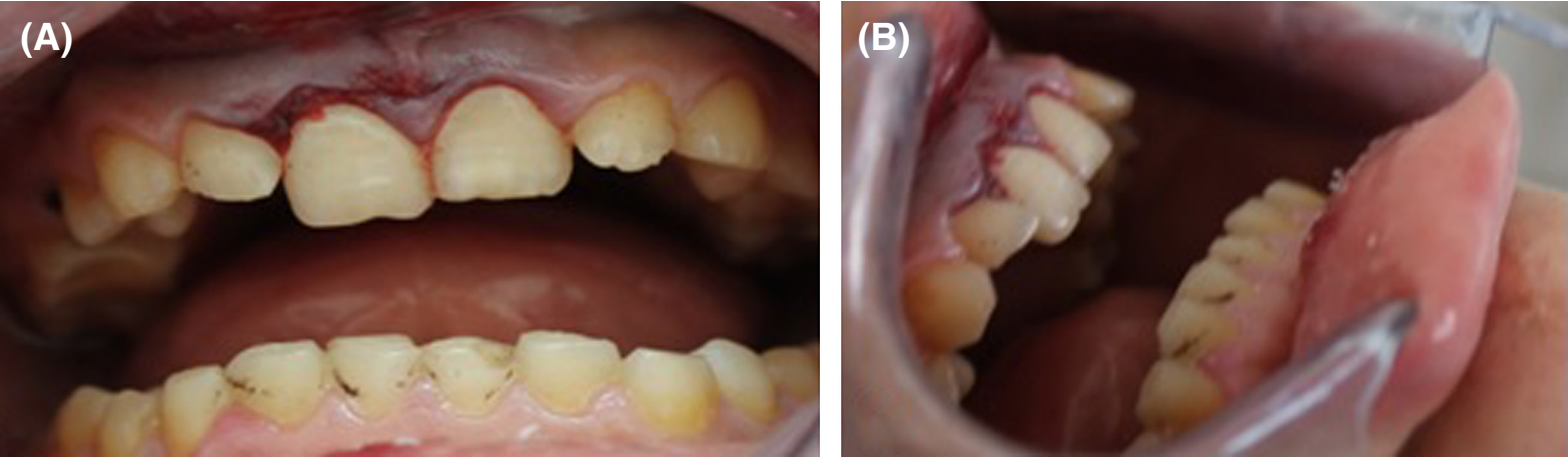
Initial intraoral view: palat

**FIGURE 2:**
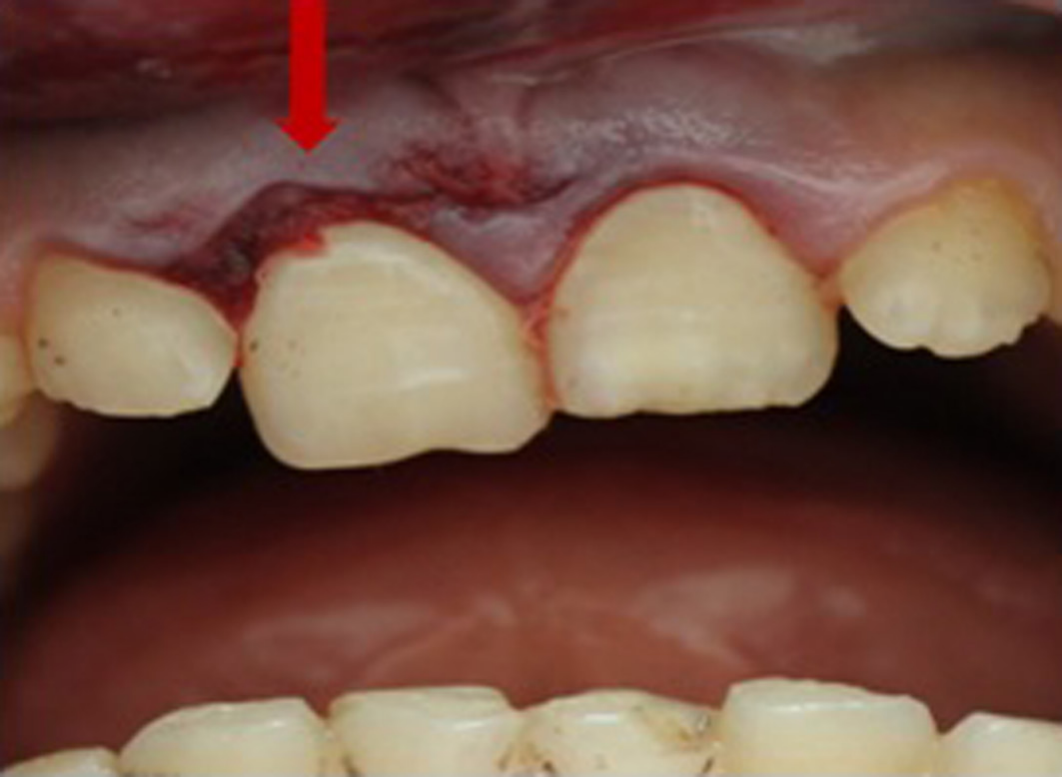
Bleeding at the gingival sulcus

A healthy 15‐year‐old patient was referred to the Department of Dentistry at the University Hospital Sahloul with trauma to his permanent maxillary incisors following a bicycle accident occurring 24 h earlier. Examination of the facial bone and the temporomandibular joint revealed no pathological signs or symptoms. However, intraoral examination showed lateral luxation of teeth 11 and 21. A displacement of the crowns of the central incisors to the palatal direction was noted in the labial and lateral views (Figure 1). It was estimated at more than 3 mm in the vestibulopalatal direction. Bleeding caused by the injury of the periodontal ligament was observed at the gingival sulcus and 4 mm above the cervical line, suggesting an associated incomplete alveolar fracture (Figure 2).

**FIGURE 3:**
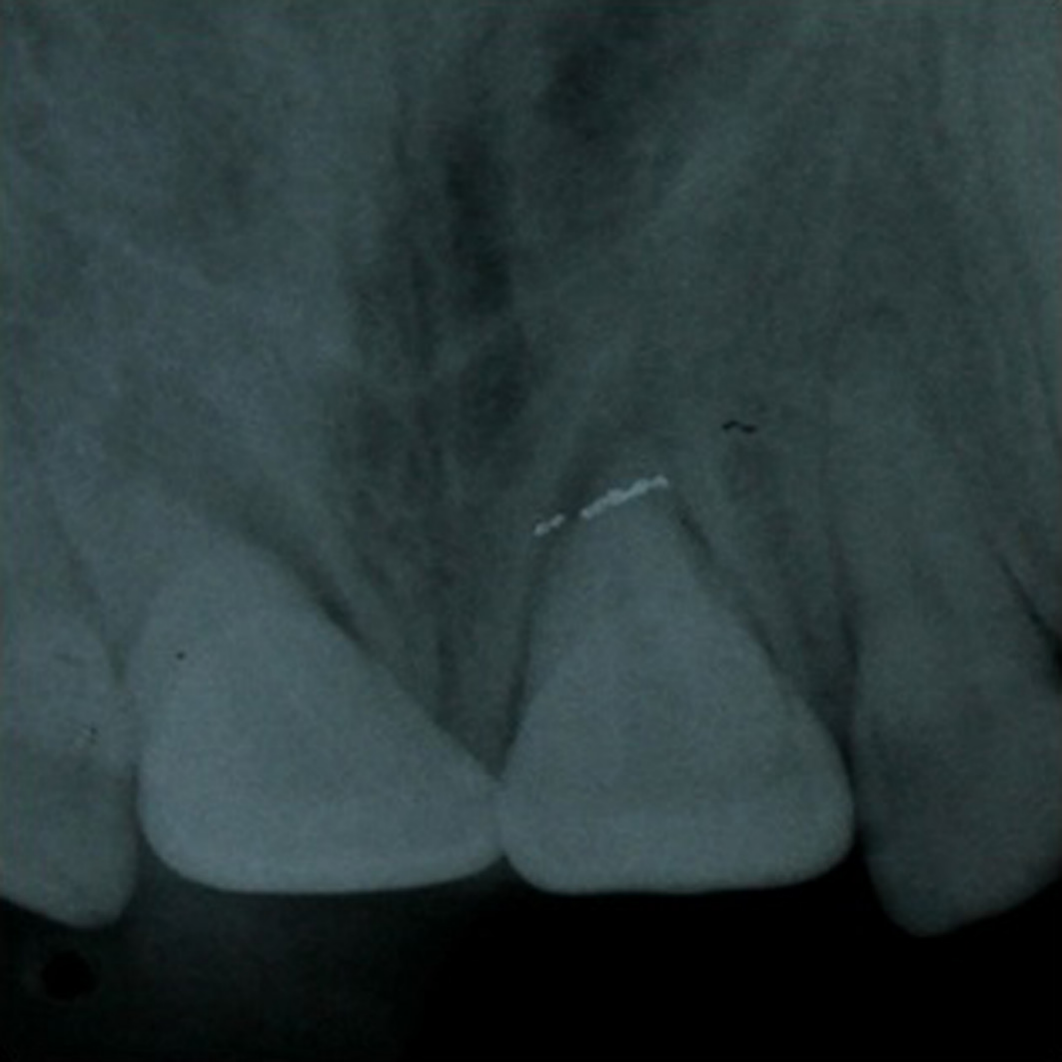
Initial periapical radiograph

At the initial examination, palpation of the bottom of the vestibule does/did not show a discontinuity of the vestibular alveolar table. Cold sensitivity was nondecisive since, according to the patient, all the anterior teeth gave diffuse responses. Percussion tests showed that traumatized teeth had a “metallic” sound. Periodontal sounding showed a pocket of 5 mm on the palatal side of the 11 and a pocket of 4 mm on the 21. Periapical radiographs excluded root fractures. They did not confirm alveolar fracture, but they showed an enlargement of the periodontal periapical space on both 11 and 21 (Figure 3).

**FIGURE 4:**
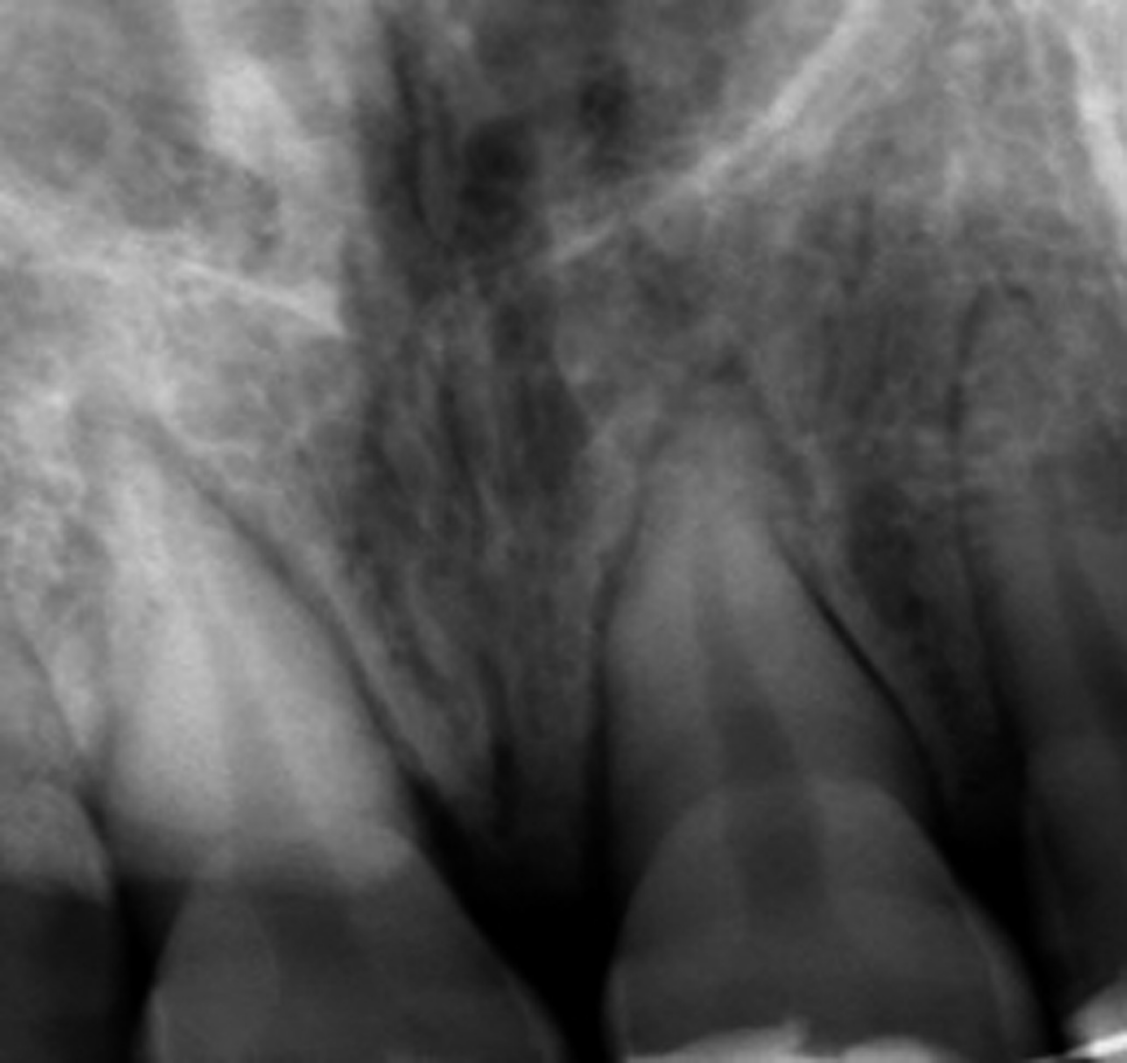
Periapical radiography after reduction

**FIGURE 5:**
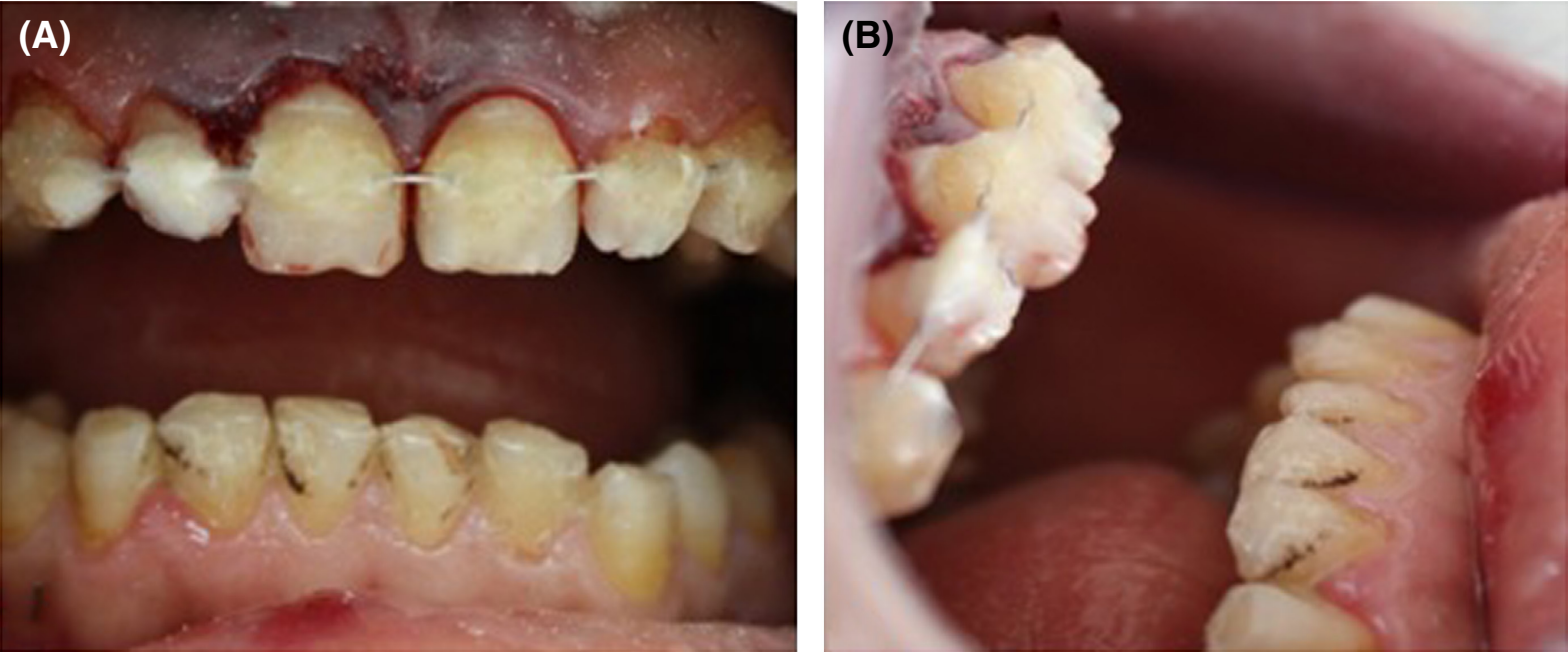
Flexible splint made with resin composite

After obtaining the parents’ informed consent, a local anesthetic was administered (1:000,000 Lidocaine). The traumatized region was generously irrigated with physiologic saline, and it was then disinfected with iodine alcohol. Both teeth were gently pushed back into their original location using the manual repositioning technique. This technique is straightforward. It uses digital pressure at the vestibular apical level along with digital pressure on the palatal side at the height of the crown. It enables the clinician to reposition the tooth to its proper position in the alveolus. Teeth were correctly repositioned into the socket. Periapical x‐ray was taken to confirm that the tooth ideal position was re‐established (Figure 4). A flexible splint was later fixed (0.4 steel wire with composite resin) from tooth 13 to tooth 23 (Figure 5a,b). It was maintained for 4 weeks.

The patient was asked to maintain proper oral hygiene using a postsurgical toothbrush. Chlorhexidine mouthwashes were also indicated to keep the affected area free of bacterial plaque since it can interfere with the healing processes.

Soft foods were recommended during the first week. An analgesic (Paracetamol 500 mg) was prescribed twice a day for 5 days together with an antibiotic (Clamoxyl 5oomg) 3 times a day for 7 days. Anti‐tetanus vaccination was checked.

In the one‐week follow‐up, the traumatized tooth was asymptomatic and it responded positively to the pulp vitality tests. No discomfort was reported, and the adjacent teeth had no signs of abnormality.

**FIGURE 6:**
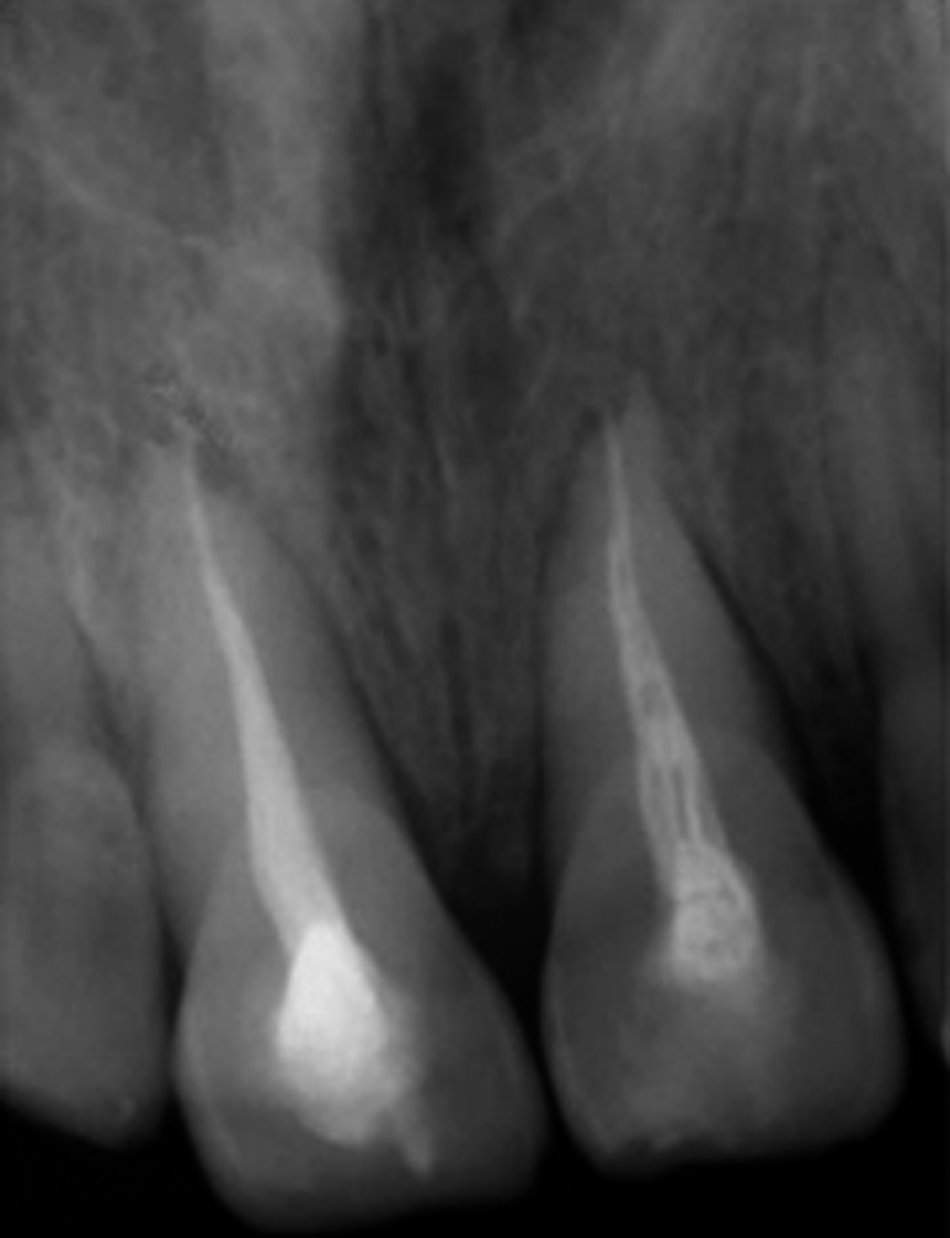
Endodontic treatment of the incisive11 and 21

At the two‐week follow‐up, the pulp vitality test was negative. Endodontic treatment was therefore indicated. Endodontic access was gained, and mechanical preparation was carefully performed (Protaper Next, Dentsply Maillefer). The root canal was irrigated at each instrument change with 2 ml of 2.5% sodium hypochlorite. Calcium hydroxide was placed in the root canal for 2 weeks, and it was followed by obturation and coronal restoration using direct composite resin (Figure 6).

**FIGURE 7:**
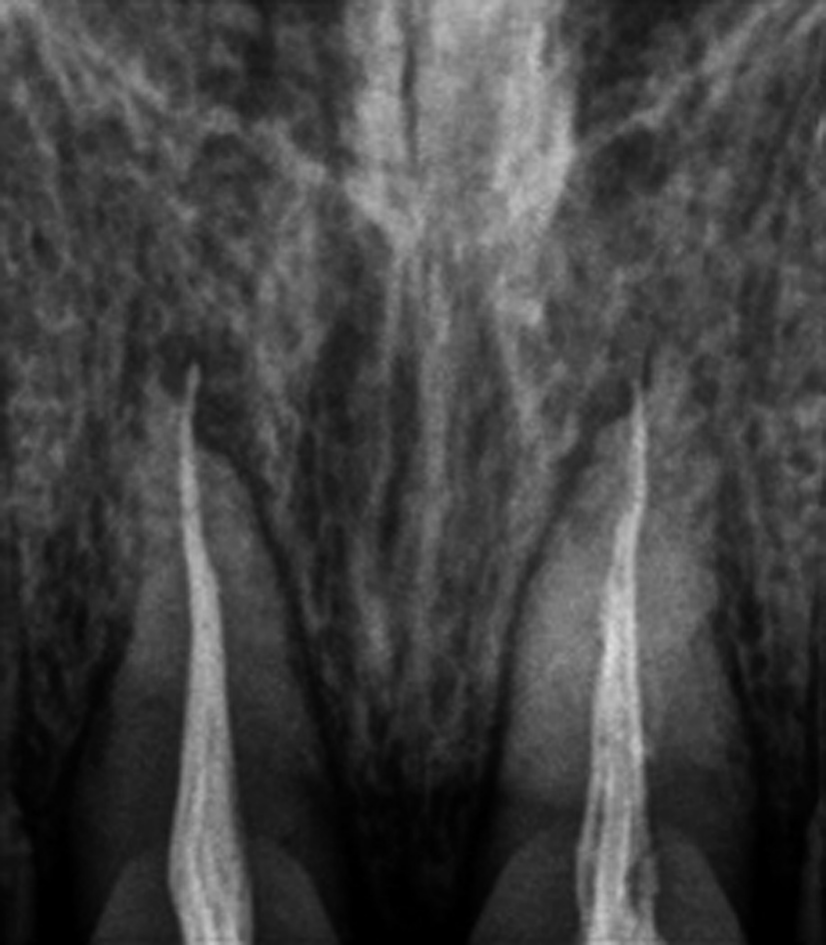
3 months follow up: Replacement resorption on teeth 11 and 21

After 3 months, no clinical symptoms of failure were observed. However, radiographic examination revealed some radiolucency in the periapical region of both teeth: replacement root resorption in the right maxillary incisor and surface root resorption in the left one (Figure 7).

## 3 DISCUSSION

A lateral luxation injury produces a displacement of the tooth in a direction other than the axial direction.^1^, ^5^ It occurs together with a fracture of the alveolar socket, and it makes the tooth immobile. The imprisonment of the root apex into the alveolar bone fracture results in tooth immobility and ankylosis percussion sound. Due to the rupture in the blood supply, pulp necrosis is common in permanent teeth with closed apex involved in the lateral luxation injury.^5^, ^6^, ^7^ External root resorption, marginal bone loss, and ankylosis, mainly in delayed or incorrect repositioning, are the most frequent periodontal complications.^7^ Lateral luxation is usually associated with injuries, such as enamel‐dentin fractures, which were not observed in the present case report.^8^

### 3.1 Diagnosis

The absence of correct diagnosis of an alveolar fracture can lead to incorrect treatment planning, resulting in complications and possible sequelae. According to IADT guidelines, CBCT is recommended based on the type and severity of the dentoalveolar injury.^9^ In the present case, the alveolar fracture was suspected based on the clinical data, but it was not possible to perform CBCT to confirm this diagnosis because of the patient's limited financial means.

### 3.2 Delay of treatment

The time elapsed between dental trauma and seeking treatment has a decisive influence on the prognosis of the injured tooth, with greater chances of success when the patient receives care within the first 30 min after injury.^10^ Dental luxation requires immediate treatment. The luxated tooth causes occlusal interference and prevents correct mouth closure. Pain can reach the condyle because of an occlusal problem.^11^ Postponing treatment can lead to inadequate tooth positioning due to the presence of organized blood clots inside the alveolar socket. Thus, immediate repositioning enables a faster and less costly resolution of the problem, responding to the treatment goals of dentoalveolar injuries.^12^, ^13^

### 3.3 Splint

Over the last decades, knowledge on the repair of traumatically displaced teeth has improved and the treatment guidelines have become more based on evidence.^13^ For instance, longer splint periods and rigid splints have been found to increase the risk of healing complications. Accordingly, flexible splints for shorter periods are more effective, as the mechanical stimulus exerted by the light movement of the teeth favors the revascularization process and is therefore capable of preventing tooth ankylosis and maintaining the vitality of Hertwig's epithelial root sheath. The splint period for periodontal ligament therapy is 2–4 weeks, and it may be prolonged to 6 or 8 weeks in cases of associated alveolar fracture.^14^

### 3.4 Monitoring

Complications may occur weeks, months, or even years after dental trauma. Thus, a long‐term follow‐up of these injuries is needed. Monitoring is required to control pulpal healing. A false‐negative response is possible until 3 months, especially for permanent immature teeth (Guidelines IADT 2012). Evaluation of pulpal health requires vitality testing (cold, electric pulp test, pulse oximeter, etc.), radiographs (periapical, panoramic, CBCT, etc.), evaluation of any symptoms (pain, swelling, etc.), and clinical monitoring for the changes in color and the development of a sinus tract, swelling, or tenderness to pressure.^15^ In the present case, a negative response to the pulp vitality test was noted only 2 weeks after trauma.

This finding can be explained by the maturity of the root canal, the delay in seeking treatment, and the severity of tooth displacement.

### 3.5 Complications

Many complications are related to severe dental traumas. The most common complications are pulp necrosis, pulp canal obliteration,^16^ root resorption (RR), infection‐related resorption, ankylosis, and marginal bone loss.^17^ RR can be classified into surface RR, inflammatory RR, and replacement RR/ankylosis, with the severity and healing process depending on factors, such as the level of root development, the extent of the damage to the periodontal tissues, and the effect of bacterial contamination of the root canal.^18^ The major complication related to severe dental trauma is replacement resorption and ankylosis. The absence of vital periodontal ligament in substantial areas of root surface may enhance resorption of the cementum and dentin by osteoclasts from the adjacent bone marrow. The resorbed tooth dentin is replaced with alveolar bone by osteoblasts.^19^

In the present case, replacement resorption was noted in the apical third of the root of 11. On the other hand, simple surface resorption was noted on 21. In fact, the greater the shock and displacement are, the higher the risk of replacement resorption following the crush and necrosis of the periodontal radicular cells is.

The type of RR determines the treatment prognosis. Replacement RR, often leading to tooth loss, is caused by necrosis of the periodontal ligament cells, thus resulting in tooth fusion to the alveolar bone.^20^ In their systematic review, Souza et al. showed that the most common type of RR reported in all the types of injuries is inflammatory RR, followed by replacement RR, surface RR, and internal RR. The progression of inflammatory RR is closely linked to the presence of infected necrotic pulp tissue and delayed endodontic treatment. However, the diagnosis of pulp necrosis is quite difficult in the first few weeks following trauma.^21^

Pulp necrosis and calcification are the most common post‐traumatic complications.^21^, ^22^ However, RR is the most concerning complication because it could lead to the loss of the traumatized tooth, mainly after luxation and avulsion.

## 4 CONCLUSION

Early diagnosis, quick and effective treatment, and short follow‐up intervals for teeth with traumatic injuries can result in better control of post‐traumatic complications, thus increasing the chances to conserve the tooth and its surrounding structures. Clinicians should therefore be aware of the incidence of RR after injuries to minimize the risk and severity of its occurrence.

## AUTHOR CONTRIBUTIONS

Ines Kallel contributed to the patient's care and follow‐up, and to writing the manuscript. Malek Lagha contributed to writing the manuscript. Eya Moussaoui contributed to the patient's follow‐up. Nabiha Douki contributed to the revision of the manuscript.

## CONFLICTS OF INTEREST

No conflicts of interest.

## ETHICAL APPROVAL

For clinical cases, the local ethics committee considers that the patient's consent is sufficient.

## CONSENT

Written informed consent was obtained from the patient to publish this report in accordance with the journal's patient consent policy.

## Data Availability

Data are available on the manuscript.
